# Age and Cytokine Gene Variants Modulate the Immunogenicity and Protective Effect of SARS-CoV-2 mRNA-Based Vaccination

**DOI:** 10.3390/vaccines11020413

**Published:** 2023-02-10

**Authors:** Letizia Scola, Donatella Ferraro, Giuseppa Luisa Sanfilippo, Simona De Grazia, Domenico Lio, Giovanni Maurizio Giammanco

**Affiliations:** 1 Clinical Pathology, Department of Biomedicine, Neuroscience and Advanced Diagnostics (Bi.N.D.), University of Palermo, Corso Tukory, 211, 90134 Palermo, Italy; 2 Microbiology, Department of Health Promotion, Mother and Child Care, Internal Medicine and Medical Specialties (PROMISE), University of Palermo, 90133 Palermo, Italy; 3 Interdepartmental Research Center “Migrate”, University of Palermo, 90133 Palermo, Italy

**Keywords:** anti-SARS-CoV-2 vaccine, anti-SARS-CoV-2 S1/S2 IgG, cytokine gene SNPs, *IL-1R1 rs2234650*, *IL-6 rs1800795*, *IL-4 rs2243250*, *TNFA rs1800629*

## Abstract

The introduction of anti-SARS-CoV-2 vaccines in late 2020 substantially changed the pandemic picture, inducing effective protection in the population. However, individual variability was observed with different levels of cellular response and neutralizing antibodies. We report data on the impact of age, gender, and 16 single nucleotide polymorphisms (SNPs) of cytokine genes on the anti-SARS-CoV-2 IgG titers measured 31 and 105 days after administration of the second dose of BNT162b2 vaccine to 122 healthy subjects from the health care staff of the Palermo University Hospital, Italy. The higher titers at 31 days were measured in the younger subjects and in subjects bearing T-positive genotypes of *IL-1R1 rs2234650* or the GG homozygous genotype of IL-6 rs1800795 SNP. T-positive genotypes are also significantly more common in subjects with higher titers at day 105. In addition, in this group of subjects, the frequency of the CT genotype of *IL-4 rs2243250* is higher among those vaccinated with higher titers. Moreover, these SNPs and *TNFA rs1800629* are differently distributed in a group of subjects that were found infected by SARS-CoV-2 at day 105 of evaluation. Finally, subjects that were found to be infected by SARS-CoV-2 at day 105 were significantly older than the uninfected subjects. Taken together, these data seem to suggest that age and polymorphisms of key cytokines, which regulate inflammation and humoral immune response, might influence the magnitude of the antibody response to vaccination with BNT162B2, prompting speculation about the possible benefit of a genetic background-based assessment of a personalized approach to the anti-COVID vaccination schedule.

## 1. Introduction

The introduction of anti-SARS-CoV-2 vaccines has substantially modified the pandemic picture, inducing effective protection in the population. Several vaccines have been developed and administered, which have shown heterogeneous efficacy and immunogenicity against the different variants of SARS-CoV-2 with wide inter-individual differences, so that booster doses were required. The Pfizer-BioNTech vaccine BNT162b2, based on lipoic nanoparticles containing the mRNA for the spike (S) protein [1], will be introduced at the end of 2020. However, it is well known that the immunogenicity and effectiveness of a vaccine can be impacted by different factors, and differential patterns of vaccine effectiveness have been observed in diverse populations, resulting from the complex interplay among the host, pathogens, and environmental factors [2,3]. Concerning the host factors, gender, aging, and genetic factors, such as the frequencies of specific genetic variants of immune inflammatory genes [4], have been largely documented. As a result, genome-wide association studies (GWAS) and other genetic investigations have focused on large panels of genes, including genetic variants of HLA, PAMPs, cytokines, chemokines, and receptor molecules, as possible causes of variability in the response to antiviral vaccines against hepatitis B, measles, rubella, influenza A, smallpox, anthrax, and mumps [5,6,7,8,9,10,11,12]. Similar results were obtained studying the association of antiviral vaccine response with polymorphic variants in cytokine genes, such as TNF-α, IL-2, IL-4 IL-10, IL-28, IFNγ [13,14,15,16]. 

However, there is currently limited evidence on factors influencing individual responses to anti-SARS-CoV-2 vaccines. A wide range of sociodemographic, biological, clinical, and nutritional factors have been reported to influence the different production of anti-spike antibodies and their titers after vaccination in people of different ages and ethnicities [17]. Therefore, individual genetic background, which influences the intensity and quality of the immune and inflammatory response, could also be implicated in the regulation of the vaccine-induced anti-SARS-CoV-2 immune response. Based on such evidence, it might be useful to evaluate the impact of cytokine gene variability on vaccine efficacy. 

Consistent with this aim, in this study we aimed to detect the biological effect of single nucleotide polymorphisms (SNPs) in cytokine genes involved in the regulation of inflammation and antibody production on the magnitude and duration of the humoral immune response induced by vaccination against SARS-CoV-2. The association of functionally relevant genetic variants in cytokine genes with anti-S1/S2 IgG antibody levels measured at 31 and 105 days after administration of the second dose of Pfizer-BioNTech’s Comirnaty BNT162b2 was evaluated.

We evaluated polymorphisms of the following IL-1 superfamily genes: IL-1A rs1800587 [18], IL-1B rs1143634 [19] and rs16944 [20], IL-18 rs187238 [21] and rs1946518 [22], which influence cytokine levels and production, IL-1RN rs315952, whose alleles modulate efficiency of inflammation control [23], and IL-1R1 rs2234650, whose alleles create two alternative putative binding sites for two different transcription factors and activation of different metabolic pathways [24]. In addition, IL-6 rs1800795 [25] and TNFA rs1800629 [26] polymorphisms, known to be involved in regulating the production of these two key cytokines for the inflammatory response, were typed. Considering the key role of IL-10 in regulating inflammation and antibody production, the IL-10 SNPs rs1800896, rs1800872, and rs3021097, which regulate IL-10 cytokine production [27], were also typed. Finally, the association of functional polymorphisms of key Th1 and Th2 cytokines (IL-4 rs2243250 [28], IL-13 rs1800925 [29], IFNG rs2430561 [30], and IFNGR2 rs2834213 [31]) with vaccination-induced anti-S1/S2 IgG antibody levels was evaluated. 

## 2. Materials and Methods

### 2.1. Subjects

Blood samples were collected at the Department of Health Promotion, Mother and Child Care, Internal Medicine, and Specialties of Excellence “G. D’Alessandro” (PROSAMI) of the University of Palermo from 122 healthy subjects (66 women and 56 men with a mean age of 49.45 ± 13.41 years) from the health care staff of the University Hospital “P. Giaccone”, professionally in contact with vulnerable people, and vaccinated with Pfizer-BioNTech’s Comirnaty BNT162b2.

All subjects, whose nasopharyngeal swab was negative for the SARS-CoV-2 biomolecular test at the time of vaccination, received the first dose of vaccine between December 2020 and July 2021 and received the second dose within the scheduled time frame (21 to 28 days after the first dose). Enrolled, vaccinated subjects underwent two blood samplings: on days 31 and 105 after the completion of the two-dose vaccination cycle. Exclusion criteria were as follows: (a) positivity for SARS-CoV-2 infection on day 31 after the second dose revealed by antigenic or serologic analyses; (b) acute or chronic diseases; and (c) drug assumption. All participants gave their informed consent. Data were encoded to ensure privacy protection of the subjects. The study was conducted in accordance with the Declaration of Helsinki and approved by the Ethical Committee of the A.O.U.P. “P. Giaccone” University Hospital (Protocol No 0006; Date 24 June 2020)

### 2.2. Quantitative Determination of Anti-SARS-CoV-2 S1/S2 IgG

Sera from the study population were collected at 31 and 105 days after the second dose of Pfizer vaccination. All samples were analyzed as previously described [32] by chemiluminescent immunoassay (CLIA) technology, (LIAISON^®^ SARS CoV-2, Diasorin, Saluggia (VC)—Italy) according to the manufacturer’s instructions on the LIAISON^®^ XL Analyzer. IgG antibodies against S1/S2 antigens of SARS CoV -2 were detected in a semi-quantitative assay with a lower limit of detection (LoD) of 0.3 AU/mL (arbitrary units/mL) and an upper limit for quantitative evaluation at 400 AU/mL. As suggested by the manufacturer, samples were considered positive when AU/mL (arbitrary unit/mL) was ≥15, and negative when AU/mL was ≤12 AU/mL, while with results between 12 and 15 AU/mL, the samples were considered borderline [33].

### 2.3. Cytokine SNP Molecular Typing

Blood specimens collected in tripotassium EDTA sterile tubes were stored at −80 °C until used for DNA extraction with the “Magna Pure” 24 System automated extraction method (Roche Diagnostics S.p.A., Monza (MB), Italy). This approach, based on a solid-phase extraction method (capture of silica-coated magnetic microspheres with high DNA affinity followed by DNA elution), allows efficient and high DNA yields. As reported in Table 1, we selected seventeen functional and common SNPs. The dbSNP NCBI database, part of the ENSEMBL project (http://www.ensembl.org/index.html, last access: 8 September 2022), was queried for the selection of SNPs. DNA samples were typed using dedicated competitive allele-specific PCR (polymerase chain reaction) assays (KASPar), based on homogeneous fluorescence resonance energy transfer (FRET) detection, developed by K-Bioscience (K-Bioscience Ltd., Hoddesdon, UK), as previously described [34]. Genotypes were determined using the 7300 system SDS software, version 1.3 (Applera Italia, MONZA (MI), Italy), on each individual sample, on the basis of the detection of fluorescence signals (single for homozygous samples, double for heterozygous samples).

### 2.4. Identification of SARS-CoV-2 Infection by Molecular Testing of Nasopharyngeal Swabs 

Samples from rhino-pharyngeal swabs were analyzed with the Allplex^tm^ SARS-CoV-2 Assay (Seegene^tm^, Arrow Diagnostics S.r.l, Genoa, Italy), which allows qualitative detection of SARS-CoV-2 by multiplex real-time RT-PCR. The assay allows simultaneous amplification and detection of the target nucleic acids of the E gene, RdRP gene, S gene, and N gene of SARS-CoV-2. The presence of specific gene sequences in the reaction is reported as a Ct value using Seegene Viewer analysis software (Seegene^tm^, Arrow Diagnostics S.r.l, Genoa, Italy). An exogenous gene is used as an internal control (IC) to monitor the entire nucleic acid extraction process and to check for PCR inhibitors.

### 2.5. Statistics

Allele and genotype frequencies were evaluated by gene count, using an online statistical analysis tool for the evaluation of SNPs (https://www.snpstats.net/start.htm, last access: 25 November 2022). The data were tested for goodness of fit between observed and expected genotype frequencies, according to Hardy-Weinberg equilibrium, by Pearson’s distribution and χ^2^ tests. Significant differences in allele, homozygous, and heterozygous genotype distributions among groups were calculated using Fisher’s exact test (adjusted for age and gender). Multiple logistic regression models were applied, using dominant (major allele homozygotes versus heterozygotes plus minor allele homozygotes) and recessive (major allele homozygotes plus heterozygotes versus minor allele homozygotes) models. Odds ratios (OR), 95% confidence intervals (95% C.I.), and p values (*p*-value cutoff < 0.05) were determined using GraphPad InStat software version 3.06 (GraphPad, San Diego, California, USA) and the above-mentioned online statistical analysis tool.

## 3. Results

### 3.1. Antibody Titers Stratified for Age and Gender 

Table 2 shows descriptive statistics of antibody titers in 122 healthy subjects treated with two doses of recombinant RNA vaccine against the SARS-CoV-2 virus, measured 31 and 105 days after administration of the second dose. In the time interval between the two doses, 39 subjects included in the study were infected with SARS-CoV-2. As reported in the table, a decrease in titers was observed in the remaining 83 uninfected subjects, comparing the values at day 31 with those at day 105. 

Since age and gender are factors that can influence the pattern of antibody response, we evaluated whether these two variables were equally distributed in the groups of subjects showing a decrease in titers above or below the median reported in Table 2. Furthermore, it was assessed whether age and gender were equally distributed in our population according to the antibody titers measured on days 31 and 105. As shown in Table 3, at day 31, subjects with titers above the group median were significantly younger than those with titers below the median. The same significant difference in age distribution was observed among uninfected subjects at day 105. Finally, subjects who had suffered through a SARS-CoV-2 infection, by day 105, despite vaccination, were significantly older than the uninfected subjects at the same time point.

Considering the results obtained, we have investigated if titer levels at 31 days were different in subjects infected compared to those that remained uninfected. We found that antibody titer levels at 31 days post-administration of the second BNT162b2 dose were significantly reduced in subjects who then developed infection, compared with those still uninfected at 105 days (Table 4).

The 66 women and 56 men included in the study showed no significant difference in the analysis by gender (Table 5). Both at 31 and 105 days post-vaccine second dose, antibody titer levels above or below the median of the group were equally distributed in men and women. No gender difference was observed at day 105 comparing SARS-CoV-2-infected versus uninfected individuals.

### 3.2. Correlation of Antibody Titers to Cytokine Gene Polymorphisms 

Subjects were classified into two groups according to whether their antibody titers were above or below the median of the titers detected at day 31 (Table 6). The complete evaluation of the effect of alleles and genotypes of the different genes on the degree of antibody response at day 31 is reported in Supplementary Table S1.

No significant results were obtained for IL-1A rs1800587, IL-1B rs1143634 and rs16944, IL-1RN rs315952, TNFA rs1800629, IL-18 rs187238 and rs1946518, IL-10 rs1800896, rs1800872, and rs3021097, IL-4 rs2243250, IL-13 rs1800925, IFNG rs2430561, and IFNGR2 rs2834213. Instead, as shown in Table 6, the CC homozygous genotype of IL-1R1 rs2234650 was less represented among subjects with a higher titer of anti-SARS-CoV-2 antibodies, whereas T-positive genotype (dominant model: C/T-T/T vs C/C, OR 4.14 (1.41–12.19) *p* 0.0071) frequency was significantly increased among these subjects. Similarly, the GG homozygous genotype of IL-6 rs1800795 SNP was more frequent among subjects with a higher titer of anti-SARS-CoV-2 antibodies, whereas C-positive genotype (associated to a reduced production of the IL-6) was correlated to lower antibody titers.

Among the 122 subjects included in the study, 39 people reported evidence of SARS-CoV-2 infection, detected by antigenic or biomolecular swab, before the second blood sampling, 105 days after the administration of the second dose of vaccine. Therefore, they were excluded from further analyses of the association of genetic polymorphisms with anti-SARS-CoV-2 S1/S2 antibody levels. Data reported in Table 7 were obtained by analyzing the cytokine gene SNP allele and genotype association with anti-SARS-CoV-2 S1/S2 antibody levels in the remaining 83 uninfected subjects. Similarly to the results obtained on days 31and 105, the CC homozygous genotype of IL-1R1 rs2234650 was less represented among subjects with a higher titer of anti-SARS-CoV-2 antibodies, whereas the IL-1R1 rs2234650 T-positive genotype was significantly increased (dominant model: OR 3.67 (1.04–12.99) *p* 0.033). Moreover, the distribution frequency of the CT genotype of the IL-4 rs2243250 SNP is higher among vaccinated individuals with higher titers. No significant results were obtained for *IL-1A rs1800587, IL-1B rs1143634* and *rs16944, IL-1RN rs315952, IL-6 rs1800795, TNFA rs1800629, IL-18 rs187238,* and *rs1946518, IL-10 rs1800896, rs1800872,* and *rs3021097, IL-13 rs1800925, IFNG rs2430561,* and *IFNGR2 rs2834213*, as shown in Supplementary Table S2.

We also assessed that most of the genotyped SNPs (*IL-1A rs1800587, IL-1B rs1143634* and *rs16944, IL-1RN rs315952, IL-18 rs187238,* and *rs1946518, IL-10 rs1800896, rs1800872,* and *rs3021097, IL-13 rs1800925, IFNG rs2430561,* and *IFNGR2 rs2834213*) were not significantly associated with susceptibility to infection in vaccinated subjects (Supplementary Table S3).

However, as shown in Table 8, the C allele of *IL-6 rs1800795* (associated with a reduced production of the cytokine) was increased in the infected subjects, mainly due to the significant rise in the frequency of distribution of the homozygous CC genotype (dominant model: G/C-C/C vs. G/G, OR 2.29 (1.05–4.96), *p* 0.045). Moreover, the group of subjects who tested positive for SARS-CoV-2 infection before day 105 was characterized by a higher frequency distribution of the heterozygous CT genotype of the *IL-1R1 rs2234650* SNP and of the homozygous AA genotype of *TNFA rs1800629*, which in Caucasians is associated with increased cytokine production. Finally, an increase in the frequency of the T allele of IL-4 rs2243250 SNP, which is associated with a greater production of cytokines, and an increase in T-positive genotypes frequency distribution (dominant model C/T-T/T vs C/C, OR 2.67 (1.21–5.88) *p* 0.014) was observed in infected subjects.

## 4. Discussion

SARS-CoV-2 infection can result in considerable variability in the severity of clinical symptoms, from asymptomatic infection to acute respiratory distress syndrome, with a clear age- and gender-dependent trend, with men older than 65 years accounting for approximately 80% of hospitalizations for severe COVID-19 [35]. Accordingly, in early 2021, COVID vaccinations were offered first to people over the age of 80, their caregivers, and sanitary staff professionally in contact with vulnerable people. Considering the impact of age and gender on severe COVID-19 susceptibility, we have evaluated the biological effect of these variables on the antibody response to the Pfizer- BNT162b2 vaccination. Our results indicate that subjects with higher antibody titers were significantly younger than those with lower titers, both at 31 and 105 days after the second vaccine dose. The same significant difference in age distribution was observed among uninfected and infected subjects at day 105. Actually, SARS-CoV-2-infected subjects were older than uninfected individuals. These results are in good agreement with data reported by other groups showing a reduction of anti-SARS-CoV-2 spike-specific IgG and neutralization titers in older subjects [36,37]. 

About 25% of the subjects recruited for this study were infected by SARS-CoV-2 between 31 and 105 days after administration of the second dose of vaccine, and age seemed to correlate with susceptibility to infection in vaccinated subjects. 

The differential gender-related susceptibility to COVID-19 is well known [38,39]. However, our data indicate that gender does not influence antibody titers or the likelihood of vaccinees acquiring a breakthrough SARS-CoV-2 infection. Banki and coworkers [40] had found no gender correlation between SARS-CoV-2-specific T- and B-cell responses, at 35 ± 8 and 215 ± 7 days after the second dose in 600 subjects who participated in a rapid mass vaccination against SARS-CoV-2 with BNT162b2 in Austria. 

Although the development of a high titer of anti-S antibodies cannot be considered the only suitable biomarker to predict protection against SARS-CoV-2 after vaccination [41,42], a lower antibody titer could be considered predictive of a higher susceptibility to infection in vaccinees [43]. Our data seem to confirm the latter, as at day 31, the antibody titers of vaccinated subjects who later developed SARS-CoV-2 infection were significantly reduced compared to those of subjects still uninfected at day 105. From our results, reduced anti-S IgG titers in subjects older than 50 years, one month after the second dose of vaccination, might be a marker for assessing the risk of SARS-CoV-2 infection. However, these findings should be confirmed in a larger group of subjects, also evaluating the cell-mediated response to anti-SARS-CoV-2 vaccines.

A key mechanism for inducing an efficient response to RNA based vaccines is the induction of a sustained inflammatory response induced by lipidic envelopment [44]. Some authors indicated that anti-S IgG and neutralizing antibody titers resulting from the second BNT162b2 dose were significantly associated with fever, a reaction in which proinflammatory cytokine activation is a mandatory mechanism [45]. Therefore, a genetically determined increase in proinflammatory cytokine production could be relevant to achieving a satisfactory rate of protection. RNA vaccines activate an inflammatory pathway mediated by IL-1 [44], which is an essential modulator in the natural and induced immune responses. We found no association between the genotypic frequency of IL-1 and an increase or decrease in the antibody titer in response to the BNT162b2 vaccine. However, a significant finding relates to the *IL-1R rs2234650* polymorphism, as *rs2234650T* carrier genotypes correspond to the highest neutralizing Ab titers at days 31 and 105, while the homozygous CC condition is associated with the lowest antibody titers. In a 2015 study, using in silico analyses, Vasilyev and coworkers [24] suggested that *rs2234650* alleles create two alternative putative binding sites for two different transcription factors. In silico structure associated to the C allele might bind yin yang 1 transcription factor (YY1), whereas the T allele structural model resulted in a binding site for the activation of protein 1 (AP-1) transcriptional factor. YY1 is involved in inflammation and tumorigenesis [26,46]. In particular, YY1 is known to be involved in viral diseases, playing a role in the activation of HIV-1 replication [47] and in the promotion of oncogenic HPV [48]. AP-1 modulates proliferation, differentiation, apoptosis, and inflammation [49] and has a central role in the induction of IL-1β production and secretion mediated by nuclear translocation of activated AP-1 [50,51]. The conformational change associated with the T allele might facilitate increased production of IL-1, which is essential to obtain protective anti-S IgG and neutralizing antibody titers toward RNA vaccines [44]. Accordingly, our data demonstrate that IL-1R1 rs2234650 T-positive genotypes are significantly associated with higher titers of anti-S IgG at both day 31 and day 105.

Antibody production by B cells is influenced by a concert of signal-specific coactivators, including inflammasome activators and IL-1 linking to the IL-1 receptor, all converging on MyD88 and associated signaling adapters. The latter lead to the activation of the NF-κB and release of trapped NF-κB/Rel transcription factors into the nucleus, resulting in the alteration of the expression of hundreds of target genes, including immunoglobulins genes [52]. 

IL-6 is involved in both activating inflammation [53] and stimulating immunoglobulin production [54,55] by driving helper lymphocyte differentiation [56]. The best known and most studied polymorphism of *IL-6* is located in the promoter, the G/C substitution at position -174 from the transcription start site (*rs1800795*). The major *-174G* allele is associated with increased mRNA expression, up to 2.4-fold following IL-1 stimulation, whereas the C allele is associated with genotypes with low production of IL-6 [23,57]. In spite of the well-known role of IL-6 in the cytokine storm induced by SARS-CoV-2 infection [58,59], no clear evidence has been presented on the role of IL-6 SNPs as susceptibility or protective factors in COVID-19. In a recent study, a haplotype common in Asia, C-T-T, represented by variant alleles of *rs1800796, rs1524107*, and *rs2066992* SNPs, associated with a reduced expression of IL-6 following inflammatory stimuli, was identified as a protective genetic background associated with a better outcome of SARS-CoV-2 infection [60]. *IL-6* promoter SNPs *rs1800795* is polymorphic almost exclusively in Caucasians, and it has been suggested that genotype *rs1800795GG*, associated with high IL-6 production, might be protective against severe COVID-19 [61,62]. Our data seem to indicate that the IL-6 rs1800795GG genotype is strongly associated with higher anti-spike antibody titers, at least at day 31 after the second dose of the BNT162b2 vaccine. On the other hand, proliferation of B-cells and their differentiation into antibody-secreting plasma cells, the key mechanism for a successful vaccine response, are sustained by type 2 T helper (Th2) cytokines such as IL-4, IL-10, and IL-6 itself [63]. In this view, a genetically determined high IL-6 production might be useful to determine a favorable environment to reach a high rate of antibody protection against SARS-CoV-2 infection, as demonstrated by other groups for vaccination against the H1N1 flu virus [64]. 

Our observation that the *IL-4 rs2243250T* positive genotype (heterozygous C/T genotype) is associated with high levels of anti-spike antibodies prompt us to hypothesize that the presence of *IL-1R1 rs2234650T*, *IL-6 rs1800795G,* and *IL-4 rs2243250T* positive genotypes, which appear to be associated with an increased production of the respective cytokines [65,66], tagged the maintenance of optimal antibody production in subjects receiving the second dose of anti-SARS-CoV-2 mRNA vaccine. 

As is well known, IFNγ is the key cytokine for IgG isotype switching, whereas IL-4 stimulates the proliferation, maturation, and differentiation of B lymphocytes in plasma cells actively secreting IgE and IgG4 [67]. In addition, IL-4 is essential to maintain naïve B cells and the production of memory B cells after exposure to an antigen or vaccination [68]. Therefore, a genetically determined increased release of IL-4 might be involved in maintaining optimal IgG production after IFNγ-mediated isotype switch.

On the other hand, when the cytokine genotype assets of subjects who had been infected after the second dose of mRNA vaccine were compared to those of uninfected subjects, we found a higher frequency of *IL-4 rs2243250 T* genotypes (associated with increased cytokine production). As is well known, IL-4, as well as IL-13, is the key cytokine in the induction of Th2- and macrophage 2 (M2)-mediated inflammation [69]. In COVID-19 patients, significantly higher IL-4 lung tissue expression and M2 macrophages were observed [70], and the prevalence of IL-4 Th2-mediated lung damage was a characteristic of the ineffective immune response elicited by SARS-CoV-2 [69]. In addition, it has been reported that in COVID-19 patients, the virus activates apoptosis by stimulating JAK-STAT6 signaling pathway through increased Th2 and IL-4 expression [71]. Therefore, it is possible to speculate that a genetic asset that favors high IL-4 production [28] may have different pleiotropic effects, such as favoring immunoglobulin production after vaccine immune stimulation or, conversely, being a susceptibility factor for COVID-19 in vaccinated subjects.

Interestingly, we found that the frequencies of *IL-1R1 rs2234650CT* and *IL-6 rs1800795C* positive genotypes were significantly increased in the group of subjects infected with SARS-CoV-2 after administration of the second dose of vaccine. The opposite genetic asset was observed in the group of subjects with the higher anti-S antibody titer at day 31, and allowed speculating that the simultaneous presence of *IL-1R1 rs2234650TT* and *IL-6 rs1800795GG* genotypes may be protective against the SARS-CoV-2 infection in vaccinated patients. Finally, a higher frequency of the homozygous AA genotype, which is characterized by increased transcriptional expression [24] of *TNFA rs1800629,* was detected in breakthrough infections. The role of TNF-α in worsening the clinical picture of COVID-19, ARDS, and systemic inflammation, is well known [72]. In addition, some research groups have identified *TNFA rs1800629A* as a marker of susceptibility to COVID-19 [73]. TNFA rs1800629G/A minor alleles seem to be associated with increased risk and severity of other viral respiratory infections, such as respiratory syncytial virus bronchiolitis and pneumonia [74]. Overall, our data highlight the complex role of genetic background in the humoral immune response against SARS CoV-2 and vaccine antigens and suggest further studies to evaluate the role of polymorphic variants following the booster vaccination cycle and in a larger population sample.

A limitation of this study is the lack of data on the cellular immune response to vaccination with BNT162b2 (e.g., evaluation of IFNγ levels by COVID-19-specific Quantiferon assay [75]), so we cannot analyze at this moment the effect of genetic background on the specific cellular immune response. Further studies are warranted to address this important topic.

## 5. Conclusions

In conclusion, our data seem to suggest that age and polymorphisms of key cytokines that regulate inflammation and humoral immune responses might influence the extent of the antibody response to the anti-SARS-CoV-2 vaccination. These data, to be confirmed in larger population samples and reevaluated at the completion of the vaccination cycle with three or four doses, could be useful for the evaluation of a personalized approach to anti-COVID vaccination scheduling. For subjects with a less effective response to the vaccine, different administration timing or a different vaccine formulation could be considered.

## Figures and Tables

**Table 1:** SNPs typed.

Gene	SNP	Position	Minor Allele	Biological Effect	References
*IL-1A*	*rs1800587*	2:112785383	T	The minor allele is associated with a greater production of the cytokine	[18]
*IL-1B*	*rs1143634*	2:112832813	T	The minor allele is associated with a greater production of the cytokine	[19]
	*rs16944*	2:112837290	A	The minor allele is associated with a reduced production of the cytokine	[20]
*IL-1RN*	*rs315952*	2:113132727	C	Minor allele is associated with an increased efficiency in inflammation control	[21]
*IL-1R1*	*rs2234650*	2:102141867	T	Alleles create two alternative putative binding site for two different transcription factors	[22]
*IL-18*	*rs187238*	11:112164265	G	The minor allele is associated with a greater production of the cytokine	[23]
	*rs1946518*	11:112164735	T	The minor allele is associated with a reduced production of the cytokine	[24]
*IL-6*	*rs1800795*	7:22727026	C	The minor allele is associated with a reduced production of the cytokine	[25]
*TNFA*	*rs1800629*	6:31575254	A	The minor allele is associated with a greater production of the cytokine	[26]
*IL-10*	*rs1800896*	1:206773552	G	The minor allele is associated with a greater production of the cytokine	[27]
	*rs1800872*	1:206773062	A	The minor allele is associated with a reduced production of the cytokine	
	*rs3021097*	1:206773289	T	The minor allele is associated with a reduced production of the cytokine	
*IL-4*	*rs2243250*	5:132673462	T	The minor allele is associated with a greater production of the cytokine	[28]
*IL-13*	*rs1800925*	5:132657117	T	The minor allele is associated with a greater production of the cytokine	[29]
*IFNG*	*rs2430561*	12:68158742	T	The minor allele is associated with a greater production of the cytokine	[30]
*IFNGR2*	*rs2834213*	21:33420603	G	The minor allele is associated with a greater production of the cytokine	[31]

**Table 2:** Anti-SARS-CoV-2 antibody titers (AU/mL) mean, standard deviation (SD), standard error (SE), median, and range (Max-Min) modifications at 31 and 105 days after the administration of the second dose of anti-SARS-CoV-2 vaccine.

	AU/mL 31 Days after 2nd Administration	AU/mL 105 Days after 2nd Administration	Percentage of Titer Decrease
Nr. of subjects	122	83	
Mean	259.95	162.39	46.37
SD	156.00	94.82	17.37
SE	14.12	10.41	1.91
Median	259.50	135.00	46.42
Max	458.00	400.00	77.73
Min	1.30	1.26	3.07

**Table 3:** Analysis of the significant differences in age distribution between different groups and subgroups of subjects.

Groups	Nr	Age ± SD	*p*
Study population	122	49.451 ± 13.419	----
Ab titer ≥ median at day 31	62	46.806 ± 13.317	0.0263
Ab titer < median at day 31	60	52.183 ± 13.077	
Uninfected with Ab titer ≥ median at day 105	41	44.341 ± 13.678	0.0226
Uninfected with Ab titer < median at day 105	42	50.786 ± 11.514	
Uninfected at day 105	83	47.602 ± 12.963	0.0258
Infected at day 105	39	53.385 ± 13.689	

**Table 4:** Anti-SARS CoV-2 antibody titer mean (AU/mL) ± standard deviation (SD) at day 31 in vaccinated subjects who then developed SARS-CoV-2 infection compared with levels detected in subjects still uninfected at 105 days post-administration of BNT162b2 second doses.

	N.	AU/mL ± SD	*p*
Infected by day 105	39	167.53 ± 149.91	<0.0001
Uninfected by day 105	83	303.38 ± 139.73	

**Table 5:** Analysis of the significant differences in gender distribution between different groups and subgroups.

Groups	Men	%	Women	%	OR (95% CI)	*p*
Study population	56	45.90	66	54.10	---	----
Ab titer ≥ median at day 31	24	19.67	38	31.15	0.55 (0.27–1.14)	0.146
Ab titer < median at day 31	32	26.23	28	22.95		
Uuninfected with Ab titer ≥ median at day 105	19	22.89	22	26.51	1.27 (0.53–3.03)	0.661
Uninfected with Ab titer < median at day 105	17	20.48	25	30.12		
Uninfected at day 105	36	29.51	47	38.53	1.37 (0.64–3.00)	0.441
Infected at day 105	20	16.39	19	15.57		

**Table 6:** Cytokine gene polymorphisms significantly affecting anti-SARS-CoV-2 S1/S2 antibody titers measured 31 days after administration of the second dose of anti-SARS-CoV-2 mRNA vaccine.

Genes and Alleles			Day 31				OR (95% CI)	*p* *
			Titer < Median		Titer ≥ Median			
			N	Freq.	N	Freq.		
*IL-1R1*	*rs2234650*	C	73	0.61	61	0.49	1.60 (0.97–2.67)	0.07
		T	47	0.39	63	0.51		
		C/C	16	0.27	5	0.08	0.24 (0.08–0.71)	0.0081
		C/T	41	0.68	51	0.82	2.00 (0.84–4.77)	0.11
		T/T	3	0.05	6	0.1	2.47 (0.56–10.88)	0.22
*IL-6*	*rs1800795*	G	75	0.62	99	0.8	0.42 (0.24–0.75)	0.003
		C	45	0.38	25	0.2		
		G/G	27	0.45	43	0.69	2.77 (1.31–5.81)	0.011
		C/G	21	0.35	13	0.21	0.49 (0.21–1.12)	0.086
		C/C	12	0.2	6	0.1	0.53 (0.18–1.57)	0.24

* adjusted by age and gender. *IL-1R1 rs2234650*: dominant model C/T-T/T Vs C/C, OR 4.14 (1.41–12.19) *p* 0.0071. *IL-6 rs1800795*: dominant model G/C-C/C vs G/G, OR 0.36 (0.17–0.76) *p* 0.017.

**Table 7:** Significant associations of genotypes of *IL-1R1 rs2234650* and *IL-4 rs2243250* with anti-SARS-CoV-2 S1/S2 antibody titers measured after 105 days from the administration of the second dose of recombinant mRNA vaccine (83 uninfected subjects).

Genes and Alleles			Day 105				OR (95% CI)	*p* *
			Titer < Median		Titer ≥ Median			
			N	Freq.	N	Freq.		
*IL-1R1*	*rs2234650*	C	50	0.6	40	0.49	1.54 (0.83–2.85)	0.212
		T	34	0.4	42	0.51		
		C/C	12	0.29	4	0.10	0.27 (0.08–0.92)	0.0495
		C/T	26	0.62	32	0.78	2.01 (0.74–5.47)	0.175
		T/T	4	0.1	5	0.12	1.60 (0.37–6.86)	0.52
*IL-4*	*rs2243250*	C	68	0.81	60	0.73	0.64 (0.31–1.33)	0.270
		T	16	0.19	22	0.27		
		C/C	30	0.71	22	0.54	0.46 (0.19–1.14)	0.115
		C/T	8	0.19	16	0.39	3.06 (1.17–8.00)	0.035
		T/T	4	0.1	3	0.07	0.81 (0.16–4.03)	0.799

* adjusted by age and gender. IL-1R1 RS2234650 C/T dominant model C/T-T/T vs C/C, OR 3.67 (1.04–12.99) *p* 0.033.

**Table 8:** Cytokine gene polymorphisms significantly associated with the detection of SARS-CoV-2 infection before day 105 post-administration of the second dose of recombinant mRNA vaccine.

Genes and Alleles			Uninfected Subjects		Infected Subjects		OR (95% CI)	*p* *
			N	Freq.	N	Freq.		
*IL-1R1*	*rs2234650*	C	90	0.54	44	0.56	0.92 (0.53–1.57)	0.784
		T	76	0.46	34	0.44		
		C/C	16	0.19	5	0.13	1.62 (0.55–4.81)	0.449
		C/T	58	0.70	34	0.87	3.33 (1.14–9.74)	0.018
		T/T	9	0.11	0	0	---	---
*IL-6*	*rs1800795*	G	128	0.77	46	0.59	2.34 1.31–4.18)	0.006
		C	38	0.23	32	0.41		
		G/G	53	0.64	17	0.44	0.44 (0.20–0.95)	0.049
		C/G	22	0.27	12	0.31	1.21 (0.52–2.83)	0.651
		C/C	8	0.1	10	0.26	2.89 (1.01–8.26)	0.048
*TNFA*	*rs1800629*	G	142	0.86	60	0.77	1.77 (0.91–3.51)	0.104
		A	24	0.14	18	0.23		
		G/G	60	0.72	25	0.64	1.46 (0.65–3.29	0.402
		G/A	22	0.27	10	0.26	0.86 (0.35–2.09)	0.732
		A/A	1	0.01	4	0.1	8.72 (0.92–82.84)	0.031
*IL-4*	*rs2243250*	C	128	0.77	49	0.63	1.99 (1.11–3.58)	0.022
		T	38	0.23	29	0.37		
		C/C	52	0.63	15	0.38	0.37 (0.17–0.82)	0.019
		C/T	24	0.29	19	0.49	2.37 (1.07–5.28)	0.033
		T/T	7	0.08	5	0.13	1.52 (0.44–5.25)	0.511

* adjusted by age and gender. IL-6 rs1800795: dominant model G/C-C/C vs G/G, OR 2.29 (1.05–4.96), *p* 0.045. IL-4 rs2243250: dominant model C/T-T/T vs C/C, OR 2.67 (1.21–5.88), *p* 0.014.

## Data Availability

All data generated or analyzed during this study are stored in electronic archives that can be supplied on reasonable request.

## Supplementary Materials

The following supporting information can be downloaded at: https://www.mdpi.com/article/10.3390/vaccines11020413/s1. Table S1: Influence of the different cytokine gene polymorphisms on the SARS-CoV-2 S1/S2 antibody titer 31 days after administration of the second dose of recombinant anti-SARS-CoV-2 mRNA vaccine; Table S2: Evaluation of the association of the alleles and genotypes of cytokine gene polymorphisms with the anti-SARS-CoV-2 S1/S2 antibody titers after 105 days from the administration of the second dose of recombinant mRNA vaccine (83 uninfected subjects); Table S3: Evaluation of the association between cytokine gene polymorphisms and the development of SARS-CoV-2 breakthrough infection within day 105 after administration of the second dose of recombinant mRNA vaccine.
