# Supplementary material for: Age and Cytokine Gene Variants Modulate the Immunogenicity and Protective Effect of SARS-CoV-2 mRNA-Based Vaccination
Source: Vaccines (Basel). 2023 Feb 10;11(2):413. doi: 10.3390/vaccines11020413 (PMC9962548; doi:10.3390/vaccines11020413)
Supplement: Supplementary file 1 [file vaccines-11-00413-s001.zip › vaccines-2160449-supplementary.pdf]

Table S1. Influence of the different cytokine gene polymorphisms on the SARS-CoV-2 S1/S2 antibody titer 31 days after administration of the second dose of recombinant anti-SARS-CoV-2 mRNA vaccine

Genes and alleles			Day 31 Titer < median		Titer ≥ median		OR (95% CI)	p*
			N	Freq.	N	Freq.		
IL-1A	rs1800587	C	77	0.64	79	0.64	1. 02 (61-1.72)	1.00
		T	43	0.36	45	0.36		
		C/C	22	0.37	21	0.34	1.13 (0.54-2.38)	0.85
		C/T	33	0.55	37	0.60	1.07 (0.51-2.26)	0.85
		T/T	5	008	4	0.06	0.94 (0.23-3.88)	0.93
IL-1B	rs1143634	C	75	0.62	88	0.71	0.69 (0.40-1.16)	0.18
		T	45	0.38	36	0.29		
		C/C	23	0.38	29	0.47	0.37 (0.35-1.45)	0.71
		C/T	29	0.48	30	0.48	0.97 (0.47-2.02	0.94
		T/T	8	0.13	3	0.05	0.32 (0.08-1.29)	0.091
	rs16944	G	76	0.63	85	0.69	0.79 (0.47-1.35)	0.42
		A	44	0.37	39	0.31		
		G/G	22	0.37	27	0.44	0.75 (0.36-1.55)	0.46
		A/G	32	0.53	31	0.5	0.91 (0.43-1.93)	0.81
		A/A	6	0.1	4	0.06	0.70 (0.18-2.68	0.6
IL-1RN	rs315952	T	84	0.7	84	0.68	1.11 (0.65-1.91)	0.78
		C	36	0.3	40	0.32		
		T/T	29	0.48	24	0.39	1.48 (0.72-3.04)	0.36
		C/T	26	0.43	36	0.58	0.46 (0.08-2.60)	0.36
		C/C	5	0.08	2	0.03	1.68 (0.80-3.52)	0.17
IL-1R1	rs2234650	C	73	0.61	61	0.49	1.60 (0.97-2.67)	0.07
		T	47	0.39	63	0.51		
		C/C	16	0.27	5	0.08	0.24 (0.08-0.71)	0.0081
		C/T	41	0.68	51	0.82	2.00 (0.84-4.77)	0.11
		T/T	3	0.05	6	0.1	2.47 (0.56-10.88)	0.22
IL6	rs1800795	G	75	0.62	99	0.8	0.42 (0.24-0.75)	0.003
		C	45	0.38	25	0.2		
		G/G	27	0.45	43	0.69	2.77 (1.31-5.81)	0.011
		C/G	21	0.35	13	0.21	0.49 (0.21-1.12)	0.086
		C/C	12	0.2	6	0.1	0.53 (0.18-1.57)	0.24
TNFA	rs1800629	G	94	0.78	108	0.87	0.54 (0.27-1.06)	0.09
		A	26	0.22	16	0.13		
		G/G	37	0.62	48	0.77	0.47 (0.21-1.04)	0.076
		G/A	20	0.33	12	0.19	0.51 (0.22-1.21)	0.12
		A/A	3	0.05	2	0.03	0.72 (0.11-4.71)	0.73
IL-18	rs 187238	G	84	0.7	94	0.76	0.75 (0.42-1.31)	0.32
		C	36	0.3	30	0.24		
		G/G	29	0.48	33	0.53	0.82 (0.41-1.67)	0.72
		C/G	26	0.43	28	0.45	1.25 (0.59-2.64)	0.56
		C/C	5	0.08	1	0.02	0.20 (0.02-1.86)	0.11
	rs1946518	G	63	0.52	69	0.56	0.82 (0.40-1.67)	0.72
		T	57	0.48	55	0.44		
		G/G	11	0.18	14	0.23	0.77 (0.32-1.86)	0.66
		G/T	41	0.68	41	0.66	0.82 (0.37-1.79)	0.62
		T/T	8	0.13	7	0.11	0.87 (0.29-2.67)	0.81
IL-10	rs1800896	A	67	0.56	55	0.44	1.59 (0.96-2.63)	0.096
		G	53	0.44	69	0.56		
		A/A	18	0.3	12	0.19	1.79 (0.77-4.13)	0.212
		A/G	31	0.52	31	0.5	0.96 (0.46-1.98)	0.902
		G/G	11	0.18	19	0.31	1.66 (0.69-3.97)	0.254

		C	94	0.78	96	0.77	1.05 (0.58-1.93)	0.882
		A	26	0.22	28	0.23		
	rs1800872	C/C	36	0.6	37	0.6	1.01 (0.49-2.09)	1.00
		A/C	22	0.37	22	0.35	1.00 (0.47-2.15)	1.00
		A/A	2	0.03	3	0.05	2.25 (0.33-15.14)	0.401
		C	85	0.71	87	0.7	1.03 (0.59-1.79)	1.00
		T	35	0.29	37	0.3		
	rs3021097	C/C	27	0.45	28	0.45	0.99 (0.49-2.03)	1.00
		C/T	31	0.52	31	0.5	0.96 (0.46-1.98)	0.901
		T/T	2	0.03	3	0.05	1.92 (0.29-12.81)	0.504
		C	81	0.68	96	0.77	0.61 (0.34-1.07)	0.087
		T	39	0.32	28	0.23		
IL-4	rs2243250	C/C	29	0.48	38	0.61	0.59 (0.29-1.21)	0.202
		C/T	23	0.38	20	0.32	0.76 (0.35-1.63)	0.483
		T/T	8	0.13	4	0.06	0.47 (0.13-1.72)	0.244
		C	91	0.76	87	0.7	1.34 (0.76-2.35)	0.387
		T	29	0.24	37	0.3		
IL-13	rs1800925	C/C	34	0.57	29	0.47	1.49 (0.73-3.04)	0.284
		C/T	23	0.38	29	0.47	1.14 (0.53-2.43)	0.743
		T/T	3	0.05	4	0.06	1.55 (0.32-7.46)	0.585
		A	69	0.57	78	0.63	0.80 (0.48-1.33)	0.433
		T	51	0.42	46	0.37		
INFG	rs2830561	A/A	14	0.23	18	0.29	0.74 (0.33-1.67)	0.539
		A/T	41	0.68	42	0.68	0.90 (0.41-1.97)	0.791
		T/T	5	0.08	2	0.03	0.43 (0.08-2.40)	0.322
		A	85	0.71	92	0.74	0.85 (0.48-1.48)	0.569
		G	35	0.29	32	0.26		
INFGR2	rs2834213	A/A	32	0.53	35	0.56	0.88 (0.43-1.80)	0.856
		A/G	21	0.35	22	0.35	0.97 (0.45-2.07)	0.933
		G/G	7	0.12	5	0.08	0.82 (0.23-2.84)	0.752

*adjusted by age and gender

IL-1R1 rs2234650: Dominant model C/T-T/T Vs C/C, OR 4.14 (1.41-12.19) p 0.0071;

IL6 rs1800795: Dominant model G/C-C/C Vs G/G, OR 0.36 (0.17-0.76) p 0.017

Table S2. Evaluation of the association of the alleles and genotypes of cytokine gene polymorphisms with the anti-SARS-CoV-2 S1/S2 antibody titers after 105 days from the administration of the second dose of recombinant mRNA vaccine (83 uninfected subjects)

Genes and alleles			Day 105				OR (95% CI)	p*
			Titer < median		Titer ≥ median			
			N	Freq.	N	Freq.		
IL-1A	rs1800587	C	55	0.65	53	0.65	1.04 (0.548-1.96)	1.00
		T	29	0.35	29	0.35		
		C/C	17	0.4	14	0.34	1.31 (0.54-3.20)	0.651
		C/T	21	0.5	25	0.61	1.50 (0.61-3.72)	0.382
		T/T	4	0.1	2	0.05	0.56 (0.09-3.38)	0.525
IL-1B	rs1143634	C	58	0.69	58	0.71	0.92 (0.47-1.79)	0.867
		T	26	0.31	24	0.29		
		C/C	21	0.5	19	0.46	1.16 (0.49-2.74)	0.827
		C/T	16	0.38	20	0.49	1.47 (0.59-3.66)	0.399
		T/T	5	0.12	2	0.05	0.36 (0.06-2.13)	0.243
	rs16944	G	55	0.65	54	0.66	0.98 (0.52-1.87)	1.00
		A	29	0.35	28	0.34		
		G/G	18	0.43	15	0.37	1.30 (0.54-3.14)	0.655
		A/G	19	0.45	24	0.59	1.46 (0.56-3.75)	0.444
		A/A	5	0.12	2	0.05	0.36 (0.06-2.13)	0.241
IL-1RN	rs315952	T	58	0.69	55	0.67	1.09 (0.57-2.10)	0.868
		C	26	0.31	27	0.33		
		T/T	19	0.45	15	0.37	1.43 (0.59-3.45)	0.505
		C/T	20	0.48	25	0.61	1.60 (0.65-3.95)	0.316
		C/C	3	0.07	1	0.02	0.40 (0.04-4.24)	0.43
IL-1R1	rs2234650	C	50	0.6	40	0.49	1.54 (0.83-2.85)	0.212
		T	34	0.4	42	0.51		
		C/C	12	0.29	4	0.10	0.27 (0.08-0.92)	0.0495
		C/T	26	0.62	32	0.78	2.01 (0.74-5.47)	0.175
		T/T	4	0.1	5	0.12	1.60 (0.37-6.86)	0.52
IL6	rs1800795	G	61	0.73	67	0.82	0.59 (0.28-1.24)	0.197
		C	23	0.27	15	0.18		
		G/G	25	0.6	28	0.68	0.2772 to 1.682	0.495
		C/G	11	0.26	11	0.27	1.14 (0.41-3.18)	0.800
		C/C	6	0.14	2	0.05	0.31 (0.06-1.70)	0.154
TNFA	rs1800629	G	70	0.83	72	0.88	0.69 (0.29-1.67)	0.509
		A	14	0.17	10	0.12		
		G/G	29	0.69	31	0.76	0.72 (0.27-1.89)	0.625
		G/A	12	0.29	10	0.24	1.00 (0.36-2.80)	1.00
		A/A	1	0.02	0	0	---	---
IL-18	rs 187238	G	59	0.7	63	0.77	0.71 (0.35-1.42)	0381
		C	25	0.3	19	0.23		
		G/G	21	0.5	23	0.56	0.78 (0.33-1.86)	0.662
		C/G	17	0.4	17	0.41	1.31 (0.51-3.36)	0.568
		C/C	4	0.1	1	0.02	0.23 (0.02-2.27)	0.164
	rs1946518	G	45	0.54	46	0.56	0.90 (0.49-1.66)	0.757
		T	39	0.46	36	0.44		
		G/G	10	0.24	9	0.22	1.11 (0.40-3.10)	1.00
		G/T	25	0.6	28	0.68	1.36 (0.53-3.46)	0.524
		T/T	7	0.17	4	0.1	0.64 (0.16-2.49)	0.519
IL-10	rs1800896	A	43	0.51	41	0.5	1.05 (0.57-1.93)	1.00
		G	41	0.49	41	0.5		
		A/A	11	0.26	10	0.24	1.10 (0.41-2.96)	1.00
		A/G	21	0.5	21	0.51	1.13 (0.46-2.78)	0.785
		G/G	10	0.24	10	0.24	0.96 (0.33-2.79)	0.943

		C	64	0.76	62	0.76	1.03 (0.51-2.10)	1.00
		A	20	0.24	20	0.24		
	rs1800872	C/C	24	0.57	23	0.56	1.04 (0.44-2.49)	1.00
		A/C	16	0.38	16	0.39	1.18 (0.47-2.99)	0.724
		A/A	2	0.05	2	0.05	1.23 (0.15-9.72)	0.851
		C	61	0.73	55	0.67	1.30 (0.67-2.53)	0.499
		T	23	0.27	27	0.33		
	rs3021097	C/C	21	0.5	17	0.41	1.41 (0.59-3.36)	0.511
		C/T	19	0.45	21	0.51	1.40 (0.57-3.45)	0.460
		T/T	2	0.05	3	0.07	1.61 (0.23-11.11)	0.625
		C	68	0.81	60	0.73	0.64 (0.31-1.33)	0.270
		T	16	0.19	22	0.27		
IL-4	rs2243250	C/C	30	0.71	22	0.54	0.46 (0.19-1.14)	0.115
		C/T	8	0.19	16	0.39	3.06 (1.17-8.00)	0.035
		T/T	4	0.1	3	0.07	0.81 (0.16-4.03)	0.799
		C	64	0.76	62	0.76	1.03 (0.51-2.10)	1.00
		T	20	0.24	20	0.24		
IL-13	rs1800925	C/C	23	0.55	23	0.56	0.95 (0.40-2.25)	1.00
		C/T	18	0.43	16	0.39	0.60 (0.23-1.61)	0.313
		T/T	1	0.02	2	0.05	2.99 (0.23-39.48)	0.39
		A	48	0.57	52	0.63	0.77 (0.41-1.43)	0.432
		T	36	0.43	30	0.37		
INFG	rs2830561	A/A	10	0.24	13	0.32	0.67 (0.26-1.77)	0.469
		A/T	28	0.67	26	0.63	0.62 (0.23-1.66)	0.333
		T/T	4	0.1	2	0.05	0.68 (0.11-4.18)	0.679
		A	62	0.74	60	0.73	1.03 (0.52-2.06)	1.00
		G	22	0.26	22	0.27		
INFGR2	rs2834213	A/A	25	0.6	22	0.54	1.27 (0.53-3.03)	0.661
		A/G	12	0.29	16	0.39	1.84 (0.69-4.92)	0.224
		G/G	5	0.12	3	0.07	0.67 (0.14-3.25)	0.615

*adjusted by age and gender

IL-1R1 RS2234650 C/T dominant model C/T-T/T Vs C/C, OR 3.67 (1.04-12.99) p 0.033;

Table S3. Evaluation of the association between cytokine gene polymorphisms and the development of SARS-CoV-2 breakthrough infection within day 105 after administration of the second dose of recombinant mRNA vaccine

Genes and alleles			Uninfected subjects		Infected subjects		OR (95% CI)	p*
			N	Freq.	N	Freq.		
IL-1A	rs1800587	C	108	0.65	48	0.62	1.16 (0.67-2.03)	0.668
		T	58	0.35	30	0.38		
		C/C	31	0.37	12	0.31	1.34 0.59-3.02)	0.545
		C/T	46	0.55	24	0.62	1.45 (0.65-3.23)	0.361
		T/T	6	0.07	3	0.08	0.90 (0.21-3.92)	0.889
IL-1B	rs1143634	C	116	0.7	47	0.6	1.53 (0.87-2.68)	0.147
		T	50	0.3	31	0.4		
		C/C	40	0.48	12	0.31	2.09 (0.94-4.68)	0.079
		C/T	36	0.43	23	0.59	1.97 (0.90-4.33)	0.087
		T/T	7	0.08	4	0.1	1.25 (0.34-4.61)	0.745
	rs16944	G	109	0.66	52	0.67	0.96 (0.54-1.69)	1.00
		A	57	0.34	26	0.33		
		G/G	33	0.4	16	0.41	0.95 (0.44-2.06)	1.00
		A/G	43	0.52	20	0.51	0.97 (0.44-2.13)	0.941
		A/A	7	0.08	3	0.08	0.81 (0.20-3.39)	0.784
IL-1RN	rs315952	T	113	0.68	55	0.71	0.89 (0.49-1.60)	0.768
		C	53	0.32	23	0.29		
		T/T	34	0.41	19	0.49	0.73 (0.34-1.57)	0.440
		C/T	45	0.54	17	0.44	0.70 (0.32-1.54)	0.383
		C/C	4	0.05	3	0.08	1.35 (0.28-6.54)	0.709
IL-1R1	rs2234650	C	90	0.54	44	0.56	0.92 (0.53-1.57)	0.784
		T	76	0.46	34	0.44		
		C/C	16	0.19	5	0.13	1.62 (0.55-4.81)	0.449
		C/T	58	0.70	34	0.87	3.33 (1.14-9.74)	0.018
		T/T	9	0.11	0	0	---	---
IL-6	rs1800795	G	128	0.77	46	0.59	2.34 1.31-4.18)	0.006
		C	38	0.23	32	0.41		
		G/G	53	0.64	17	0.44	0.44 (0.20-0.95)	0.049
		C/G	22	0.27	12	0.31	1.21 (0.52-2.83)	0.651
		C/C	8	0.1	10	0.26	2.89 (1.01-8.26)	0.048
TNFA	rs1800629	G	142	0.86	60	0.77	1.77 (0.91-3.51)	0.104
		A	24	0.14	18	0.23		
		G/G	60	0.72	25	0.64	1.46 (0.65-3.29	0.402
		G/A	22	0.27	10	0.26	0.86 (0.35-2.09)	0.732
		A/A	1	0.01	4	0.1	8.72 (0.92-82.84)	0.031
IL-18	rs 187238	G	122	0.73	56	0.72	1.09 (0.59-1.99)	0.877
		C	44	0.27	22	0.28		
		G/G	44	0.53	18	0.46	1.32 (0.61-2.82)	0.561
		C/G	34	0.41	20	0.51	1.39 (0.64-3.04)	0.412
		C/C	5	0.06	1	0.03	0.35 (0.04-3.20)	0.331
	rs1946518	G	91	0.55	41	0.53	1.09 (0.64-1.88)	0.784
		T	75	0.45	37	0.47		
		G/G	19	0.23	6	0.15	1.63 (0.59-4.48)	0.471
		G/T	53	0.64	29	0.74	1.79 (0.75-4.26)	0.178
		T/T	11	0.13	4	0.1	0.69 (0.20-2.38)	0.555

IL-10		A	84	0.51	38	0.49	1.08 (0.63-1.85)	0.891
		G	82	0.49	40	0.51		
	rs1800896	A/A	21	0.25	9	0.23	1.3 (0.4692.76)	1.00
		A/G	42	0.51	20	0.51	1.00 (0.46-2.17)	0.994
		G/G	20	0.24	10	0.26	1.28 (0.51-3.19)	0.599
		C	126	0.76	64	0.82	0.69 (0.35-1.36)	0.323
		A	40	0.24	14	0.18		
	rs1800872	C/C	47	0.57	26	0.67	0.65 (0.29-1.45)	0.327
		A/C	32	0.39	12	0.31	0.66 (0.29-1.52)	0.322
		A/A	4	0.05	1	0.03	0.39 (0.04-3.82)	0.388
		C	116	0.7	56	0.72	0.91 (0.50-1.65)	0.881
		T	50	0.3	22	0.28		
	rs3021097	C/C	38	0.46	17	0.44	1.09 (0.51-2.35)	0.848
		C/T	40	0.48	22	0.56	1.37 (0.63-2.98)	0.425
		T/T	5	0.06	0	0	---	---
IL-4	rs2243250	C	128	0.77	49	0.63	1.99 (1.11-3.58)	0.022
		T	38	0.23	29	0.37		
		C/C	52	0.63	15	0.38	0.37 (0.17-0.82)	0.019
		C/T	24	0.29	19	0.49	2.37 (1.07-5.28)	0.033
		T/T	7	0.08	5	0.13	1.52 (0.44-5.25)	0.511
IL-13	rs1800925	C	126	0.76	52	0.67	1.57 (0.87-2.84)	0.164
		T	40	0.24	26	0.33		
		C/C	46	0.55	17	0.44	1.61 (0.75-3.46)	0.248
		C/T	34	0.41	18	0.46	1.53 (0.67-3.46)	0.314
		T/T	3	0.04	4	0.1	2.93 (0.61-14.12)	0.182
INFG	rs2830561	A	100	0.6	47	0.6	0.99 (0.58-1.73)	1.00
		T	66	0.4	31	0.4		
		A/A	23	0.28	9	0.23	1.28 (0.53-3.10)	0.663
		A/T	54	0.65	29	0.74	1.68 (0.71-3.97)	0.233
		T/T	6	0.07	1	0.03	0.27 (0.03-2.40)	0.177
INFGR2	rs2834213	A	122	0.73	55	0.71	1.16 (0.64-2.11)	0.646
		G	44	0.27	23	0.29		
		A/A	47	0.57	20	0.51	1.24 (0.58-2.66)	0.697
		A/G	28	0.34	15	0.38	1.28 (0.57-2.85)	0.551
		G/G	8	0.1	4	0.1	0.91 (0.25-3.31)	0.884

*adjusted by age and gender

IL6 rs1800795: Dominant model G/C-C/C Vs G/G, OR 2.29 (1.05-4.96) p 0.045

IL-4 rs2243250: Dominant model C/T-T/T Vs C/C, OR 2.67 (1.21-5.88) p 0.014
